# Quantitative analysis of *Streptococcus mutans*, *Bifidobacterium*, and *Scardovia Wiggsiae* in occlusal biofilm and their association with Visible Occlusal Plaque Index (VOPI) and International Caries Detection and Assessment System (ICDAS)

**DOI:** 10.1007/s40368-024-00962-y

**Published:** 2024-12-23

**Authors:** P. Thitisakyothin, S. Chanrat, R. L. Srisatjaluk, K. Mitrakul

**Affiliations:** 1 Department of Pediatric Dentistry, Faculty of Dentistry, Mahidol University, 6 Yothee Street, Ratchathewi, Bangkok, 10400 Thailand; 2 Department of Pediatric Dentistry, College of Dental Medicine, Rangsit University, Pathum Thani, Thailand; 3 Department of Oral Microbiology, Faculty of Dentistry, Mahidol University, 6 Yothee Street, Ratchathewi, Bangkok, 10400 Thailand

**Keywords:** Occlusal biofilm, *S. mutans*, *S. wiggsiae*, *Bifidobacterium*, Real-time PCR

## Abstract

**Aims:**

To quantitatively detect *S. mutans*, *Bifidobacterium,* and *S. wiggsiae* in occlusal biofilm from permanent first molars based on the Visible Occlusal Plaque Index (VOPI), and to analyse the association between their levels and the occlusal enamel caries occurrence following the diagnosis of the International Caries Detection and Assessment System (ICDAS).

**Study design:**

One hundred twenty plaque samples were collected from children aged 6–8 years and divided into four groups (*n* = 30 each group) according to VOPI scores (0 = no visible plaque, 1 = thin plaque, 2 = thick plaque, and 3 = heavy plaque). Scores 0 and 1 were identified by running dental probe on the groove. Scores 2 and 3 were visually identified. ICDAS scores were recorded by scoring 0–3 (0 = sound tooth surface, 1 = opacity or discoloration of enamel after air drying, 2 = visual change in enamel when wet, and 3 = localised enamel breakdown).

**Methods:**

DNA was extracted from plaque samples and performed quantitative real-time PCR using SYBR green and specific primers for total bacteria including the 16S rRNA gene sequences conserved in all bacteria (BAC16S), *S. mutans*, *Bifidobacterium,* and *S. wiggsiae.*

**Results:**

Ages of the children were different amongst VOPI groups (*p* < 0.001). Levels of total bacteria (*p* < 0.001) and *S. mutans* (*p* = 0.026) increased when VOPI increased. The ratio of *S. mutans* to total bacteria (*p* = 0.015) and the ratio of *Bifidobacterium* to total bacteria (*p* < 0.001) decreased from VOPI 0 to VOPI 3. Significant differences in total bacteria (*p* < 0.001) and *S. mutans* (*p* = 0.018) were detected from VOPI 0 to VOPI 2. A difference in *Bifidobacterium* (*p* < 0.001) was detected from VOPI 0 to VOPI 1.

**Conclusion:**

Quantities of total bacteria (*p* < 0.001), *S. mutans* (*p* = 0.02) and ICDAS scores (*p* < 0.001) and VOPI scores were positively correlated. Quantities of ratio of *S. mutans* to total bacteria (*p* = 0.003) and ratio of *Bifidobacterium* to total bacteria (*p* < 0.001) and VOPI scores and ICDAS scores (*p* < 0.001) were negatively correlated.

## Introduction

Dental caries remains the most prevalent diseases worldwide (da Costa Rosa et al. 2021). It is a multifactorial disease and belongs to the group of complex common diseases (da Costa Rosa et al. 2021). Approximately 2.43 billion people worldwide have dental caries in their permanent teeth. In Thailand, the prevalence of dental caries in 12-year-old children was recently 53%, with especially high caries detection on their first permanent molars (National oral health survey 2023). Dental caries is provoked by the disbiose caused in biofilm or dental plaque which comprise more than 800 species of microorganisms living together due to frequent sugar intake (Fejerskov 2004). The development of caries lesions is localised to susceptible areas for biofilm accumulation, i.e. in the pits and fissures on occlusal surfaces and proximal to the gingival margin on smooth surfaces (Nyvad et al. 2013; Carvalho 2014). Biofilm accumulation is also enhanced during tooth eruption due to reduced mechanical oral function and a reduction in cleaning efficiency, leading to a higher caries experience on molars (Carvalho et al. 1991, 2009).

Much effort has been made to study and analyse the bacterial composition in caries lesions biofilm, especially in relation to occlusal caries. Occlusal surfaces continue to carry a major burden of caries worldwide (Schwarz et al. 1994; Brown and Selwitz 1995). Therefore, studies exploring the ecology of occlusal caries are highly recommended. Previous studies of the microbiology of occlusal caries used a classical cultivation of the bacteria and mainly focussed on mature plaque ecology. Those studies have contributed to important information about the predominant composition of the biofilm in sound and carious occlusal surfaces, but do not include yet uncultured organisms (Mikkelsen et al. 2000; Thurnheer et al. 2001). In addition, site-specific sampling of occlusal caries lesions has already been performed and investigated by up-to-date molecular techniques, but the studies have been limited to only advanced stages of caries lesions involving the dentin (Mantzourani et al. 2009a, b; Lima et al. 2011). There is a significant knowledge gap in bacterial composition within the incipient stage of caries lesion progression and the ecology of caries in occlusal caries and biofilm. Recently, the oralome and its dysbiosis theory have summarised dynamic interactions between the ecological community of oral microorganisms and the host (Radaic and Kapila 2021). These microorganisms form a complex ecosystem that thrives in the dynamic oral environment. Interspecies and host–microbe interactions substantially influence the microbial composition, which in turn can impact the health and disease status of the host. Children who have severe early childhood caries (S-ECC) demonstrate elevated levels of various genera, including *Streptococcus mutans, Bifidobacterium* and *Scardovia wiggsiae. S. mutans* has been extensively studied due to its cariogenic characteristics and is currently recognised as one of the primary pathogens linked to dental caries (Mitrakul et al. 2017; Tantikulchan et al. 2022; Dhamnernsawat et al. 2024). Previous study showed that *S. mutans* was observed on sites with both active and inactive caries, whereas *Bifidobacterium* was only detected in active caries and was not frequent residents inside the shallow fissures.

*S. mutans* is a commonly isolated microorganism from dental plaque (Tanner et al. 2011; Tantikulchan et al. 2022; Mitrakul et al. 2017). Previous studies have shown an association between *S. mutans* and S-ECC children and are used as one of the microbial parameters for assessing children’s caries risk (Tanzer et al. 2001; Tanner et al. 2011). Recent studies in Thai children found that *S. mutans* in plaque was higher in S-ECC children (Mitrakul et al. 2017; Tantikulchan et al. 2022). Other species have been recognised that are significantly associated with S-ECC when *S. mutans* is not detected, including *Bifidobacterium* and *S. wiggsiae* (Valdez et al. 2016; Mitrakul et al. 2017; Tantikulchan et al. 2022; Dhamnernsawat et al. 2024).

*Bifidobacterium* is anaerobic, Gram-positive, and rod-shaped. It has been isolated from saliva, dental plaque and dentinal caries (Valdez et al. 2016; Modesto et al. 2006; Mantzourani et al. 2009a, b; Nair et al. 2017). *Bifidobacterium* were shown to be possibly cariogenic due to its capacity to generate an acidic environment, resistance to low pH, and promotion of biofilm formation when co-adhered with a primary coloniser (Modesto et al. 2006). Previous studies in Thai children also reported that *Bifidobacterium* levels were significantly higher in the supra gingival plaque of S-ECC children when compared with caries-free children (Mitrakul et al. 2017; Dhamnernsawat et al. 2024).

*S. wiggsiae* is an anaerobic Gram-positive bacillus. In vitro studies show that *S. wiggsiae* growth and acid tolerance are similar to *S. mutans* (Henne et al. 2015). Previous studies have found that *S. wiggsiae* was detected in *S. mutan*s-negative samples, which suggests that *S. wiggsiae* might be a secondary aggressor and implicated with caries progression at a later stage of disease when *S. mutans* is not the main pathogenic specie (Henne et al. 2015). Furthermore, some studies found an association with the combination of *Bifidobacterium, S. wiggsiae* and *S. mutans* with caries, and this might be valuable in caries risk assessment (Valdez et al. 2016; Mitrakul et al. 2017; Tantikulchan et al. 2022; Dhamnernsawat et al. 2024)*. S. wiggsiae* was associated with advanced dentinal caries on occlusal surfaces in young children, initial white spot lesions in older children and dentinal caries in adults (Henne et al. 2015; Tanner et al. 2016).

Few indices are available in the literature for research on occlusal biofilm. The Visible Occlusal Plaque Index (VOPI) was developed to assess the occurrence and distribution of occlusal biofilm in relation to caries (Carvalho et al. 1989). Longitudinal studies investigating the occurrence and distribution of occlusal biofilm during tooth eruption in relation to caries by means of the VOPI showed that erupting occlusal surfaces favoured the accumulation of thick and heavy plaque due to their limited mechanical oral function and difficulties associated with tooth brushing. A higher incidence of active lesions was observed in erupting occlusal surfaces in contrast with fully erupted surfaces that mainly presented no or thin plaque scores and inactive lesions (Carvalho et al. 1991, 2009). Use of the VOPI is recommended as an additional clinical tool to estimate caries lesion activity along with the clinical characteristics of the lesions, and to support treatment decisions in daily practice.

ICDAS is a clinical scoring system that is used to detect and assess dental caries. It was generated to be used in dental education, clinical applications and researches (Dikman et al. 2015). This scoring system can be used on coronal surfaces and root surfaces and can be applied for enamel caries, dentine caries, non-cavitated lesions and cavitated lesions to detect and assess these lesions (Dikman et al. 2015). Dental caries status was recorded on a 0–6 scale, which ranges from sound tooth to enamel breakdown.

The aim of this study was to quantitatively detect *S. mutans*, *Bifidobacterium* and *S. wiggsiae* in occlusal biofilm from permanent first molars obtained from Thai children based on the Visible Occlusal Plaque Index (VOPI), and to analyse their association between levels of those bacteria and the occurrence of occlusal enamel caries in each group following the diagnosis protocol of the International Caries Detection and Assessment System (ICDAS).

## Materials and methods

This is a cross-sectional study. The study protocol was approved by the Human Institutional Review Board of the Faculty of Dentistry and the Faculty of Pharmacy, Mahidol University (MU-DT/PY-IRB 2023/045.1508). A statistician consultation was done based on previous studies, performed with α = 0.05 and power of 80%, using the software package Primer of Biostatistics (McGraw-Hill, NY, USA), (Tantikulchan et al. 2022). A minimum of 30 children in each group was enough to achieve statistical difference.

### Subject selection

Thai children aged 6 to 8 years old were recruited from Chumchon Prachathipat Wittayakhan School, Pathumthani Province, Thailand. Consent forms were signed. We selected 6 to 8-year-old children as subjects for plaque collection because of the first permanent molars are erupting around this age. Longitudinal studies investigating the occurrence and distribution of the occlusal biofilm during tooth eruption in relation to caries by means of the VOPI showed that erupting occlusal surfaces favoured the accumulation of thick and heavy plaque due to their limited mechanical oral function and difficulties associated with tooth brushing. Simultaneously, a higher incidence of active lesions was observed in erupting occlusal surfaces in contrast with fully erupted surfaces (Carvalho et al. 1991, 2014).

A full mouth clinical examination was performed using World Health Organisation criteria (Ismail et al. 2009) and the diagnosis protocol of the ICDAS and the VOPI (see below). Subjects were screened for identification of those with first permanent molars without occlusal dentin caries, occlusal fillings or occlusal sealants. Subjects who had any systemic diseases, were taking any kind of antibiotics, had received professional fluoride application, had undergone any dental treatment within 2 months prior to the sample collection period or had partially erupted first permanent molars with more than 1/4 of the occlusal surfaces covered with gingival tissue were excluded.

### Clinical examination

Two examiners (PT and PA) who were in a residency training programme in Paediatric Dentistry were trained and calibrated prior to performing a clinical examination. The agreement for VOPI was substantial (Weighted Kappa = 0.91), as was the agreement for ICDAS (Weighted Kappa = 0.83).

**Fig. 1:**
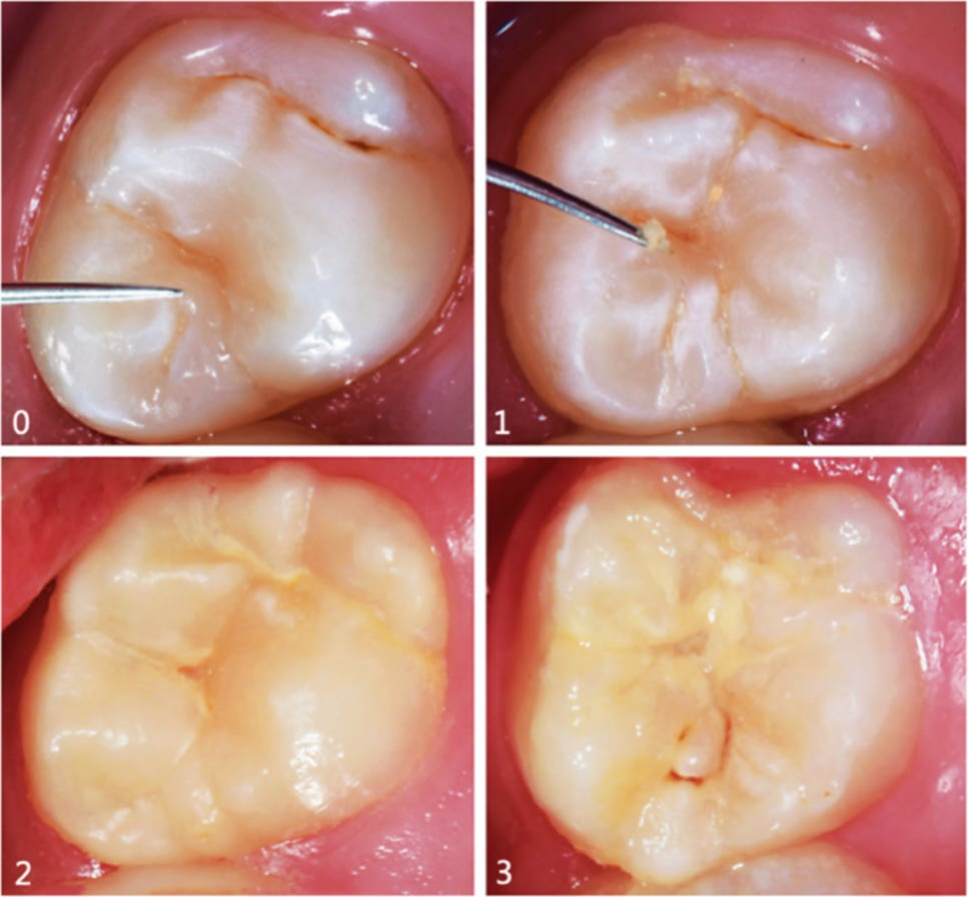
Clinical pictures illustrating the VOPI scores (23)

**Fig. 2:**
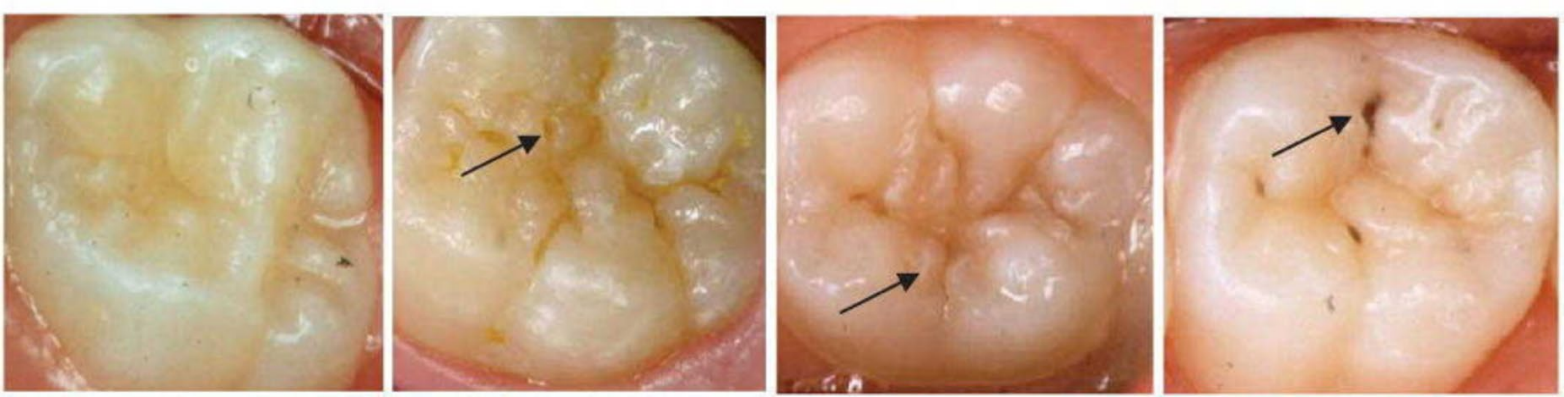
Clinical pictures illustrating the ICDAS scores (26)

Plaque samples were categorised into four groups according to VOPI scores and ICDAS scores. The total sample consisted of 120 teeth (*n* = 30 teeth in each group). VOPI was recorded (Fig. 1). Four scores of the VOPI are as follows: 0 = no visible plaque identified when carefully running a dental probe on the groove fossa-system, 1 = thin plaque: hardly detectable plaque which is restricted to the groove-fossa-system and identified by carefully running a dental probe on the groove-fossa-system, 2 = thick plaque: easily detectable plaque on the groove-fossa-system identifiable with the naked eye, 3 = heavy plaque: occlusal surfaces partially or totally covered with heavy plaque accumulation identifiable with the naked eye (Carvalho et al. 2016). Recorded ICDAS scores of permanent first molars by scoring 0–3 which were—0 = sound tooth surface: no evidence of caries after 5 s of air drying; 1 = first visual change of enamel opacity or discoloration (white or brown) is visible at the entrance to the pit or fissure seen after prolonged air drying; 2 = distinct visual change in enamel visible when wet, lesion must be visible when dry; and 3 = localised enamel breakdown (without clinical visual signs of dentinal involvement) seen when wet and after prolonged air drying (Fig. 2).

### Plaque sample collection

Occlusal dental plaque was collected using a sterile toothpick by gently dragging a toothpick on the occlusal groove of permanent first molars which was carefully isolated by multiple sterile gauzes until dry area around molars were obtained to prevent the saliva contamination. Plaque was released from a toothpick in 1 ml of TE buffer. All samples were immediately transported on ice to the Oral Biology Laboratory and stored at  –20℃ until the DNA extraction process.

### DNA extraction

DNA was extracted based on enzymatic lysis using a commercial kit (Flavogen, Taiwan) as previously described (Mitrakul et al. 2017). In brief, 20 µl of Proteinase K was added, along with 400 µl of FABG buffer and 20 µl of a lysozyme mixture (lysozyme 20 mg/ml and mutanolysin (Sigma Aldrich, USA) in 1:10 proteinase K) and vortexed. This was incubated at 60 °C for 1 h. 200 µl ethanol was added and centrifuged at 11,000 rpm for 30 s. The solution was transferred into a spin column and centrifuged for 1 min. The supernatant was discarded, 500 µl of W1 buffer was added and centrifuged for 1 min. The supernatant was discarded. Then 750 µl of wash buffer was added and centrifuged for 1 min. The next step was adding 50 µl of elution buffer, left at room temperature for 3 min, before a final centrifuge for 2 min. The extracted DNA concentration and purity were measured using a spectrophotometer at 260 nm/280 nm (Nanodrop 2000C Thermo Scientific, Delaware, USA).

### Culture condition and standard strains

Two bacterial strains (*Bifidobacterium longum* (subspecies 51,139) and *S. mutans (*ATCC 25175) were used as standard strains.

*Bifidobacterium longum* (subspecies 51,139) was grown on MRS broth supplemented with 0.05%(w/v) L-cysteine HCl and was anaerobically (5% CO_2_) incubated at 37 °C for 48 h. *S. mutans* was grown anaerobically (5% CO₂) in Brain Heart Infusion broth at 37 °C for 24–48 h. Gram stain was done prior to the genomic DNA extraction. A tenfold serial dilution, starting from 10^8^–10^2^ CFU/ml, was performed.

### Quantitative real-time PCR

Using specific primers (Table 1), the reaction mixture (total volume of 20 μl) contained (varied from 2–9.1 μl) water, 10 μl of 2X KAPA SYBR^®^ FAST qPCR Master Mix, 0.4 μl of 10 μM forward and reverse primer, and (varied from 0.1–7.2) μl of bacteria DNA. The thermocycler (C1000^™^ Thermal cycler and CFX 96 Real-time System) was set for 40 cycles. Each cycle consisted of enzyme activation at 95 °C for 3 min, denaturing at 95 °C for 3 s, annealing at 55.9 °C, 55.9 °C, 51 °C and 53 °C for 20 s for universal BAC16S, *S. mutans*, *Bifidobacterium* and *S. wiggsiae*, respectively. Melting curves were generated from 60 °C to 95 °C and read every 0.5 °C for 5 s.

**Table 1:** Primers used in this study

Primer name		Nucleotide sequence (5′ to 3′)	Expected amplicon (base pair, bp)	References
Universal BAC16S	F	5′-TGG AGC ATG TGG TTT AAT TCG A-3′	160	Sinsimer et al. 2005
	R	5′-TGC GGG ACT TAA CCC AAC A-3′		
*S. mutans*	F	5-AGC CAT GCG CAA TCA ACA GGT T-3′	415	Yano et al. 2002
	R	5-CGC AAC GCG AAC ATC TTG ATC AG-3′		
*Bifidobacterium*	F	5′ CTC CTG GAA ACG GGT GG-3	550	Matsuki et al. 2004
	R	5′ GGT GTT CTT CCC GAT ATC TAC A-3′		
*S. wiggsiae*	F	5′-GTG GAC TTT ATG AAT AAG C-3′	200	Tanner et al. 2011
	R	5′-CTA CCG TTA AGC AGT AAG-3′		

### Agarose gel electrophoresis

**Fig. 3:**
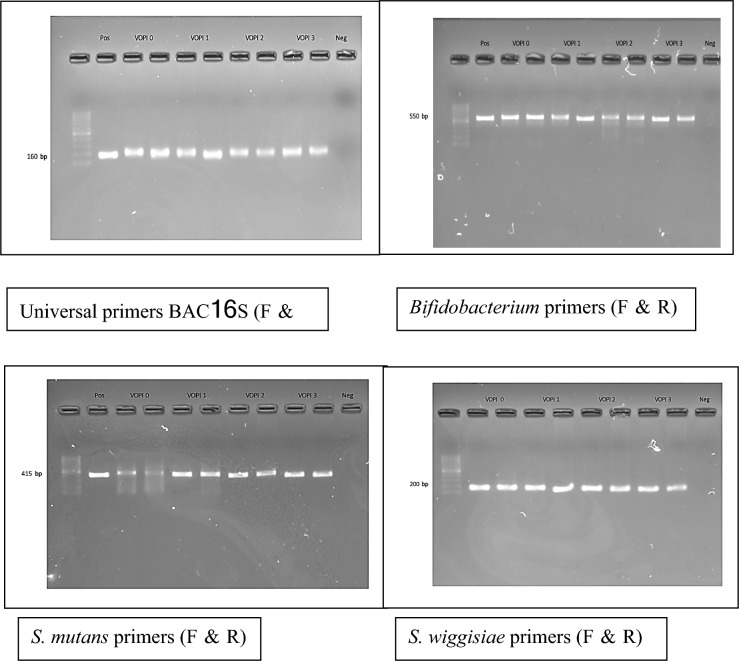
Agarose gel electrophoresis of real-time PCR product using universal primers BAC16S, and specific primers for *S. mutans*, *Bifidobacterium* and *S. wiggsiae*

Amplified PCR products were checked with 2% agarose gel (UltraPure Agarose, ThermoFisher Scientific, USA). This part was used to identify specificity and sensitivity of each primer (Fig. 3). Gel images were captured with a digital imaging system (Molecular Imager ®Gel docTM Systems, Bio-Rad Laboratories Inc., CA, USA).

### Statistical analysis

All data were recorded and analysed by IBM SPSS Statistics for Windows, Version 28.0 (IBM SPSS Statistics for Windows, Version 28.0. Armonk, NY, USA: IBM Corp.) Normality of the data was tested by Kolmogorov–Smirnov and Shapiro–wilk tests. The different levels of bacteria amongst VOPI groups were tested by Mann–Whitney U test. Analysis of median quantities of bacteria amongst VOPI groups was tested by non-parametric Kruskal–Wallis H test. Correlation analysis between the quantities of bacteria, VOPI and ICDAS was tested by spearman’s rho correlation coefficient. A value of *p* < 0.05 was accepted as statistically different.

## Results

The ages of the children were significantly different between the VOPI groups (*p* < 0.001). Mean ± standard deviation (SD) of children’s age was 6.71 ± 0.21, 6.83 ± 0.22, 7.05 ± 0.24 and 7.12 ± 0.26 in VOPI 0, 1, 2 and 3, respectively. Gender and the location of permanent molars did not show statistically significant differences (Table 2).

**Table 2:** Mean age, gender and location of permanent molars of subjects in four VOPI groups

Variables		VOPI 0	VOPI 1	VOPI 2	VOPI 3	*p* value
Children’s age (years)	Mean ± SD	6.71 ± 0.21	6.83 ± 0.22	7.05 ± 0.24	7.12 ± 0.26	< 0.001*
	Median (IQR)	6.7 (0.3)	6.8 (0.2)	7 (0.4)	7.1 (0.4)	< 0.001*
Children’s genders (*n*%)						0.947
Male		16 (53.3)	15 (50)	14 (46.7)	15 (50)	
Female		14 (46.7)	15 (50)	16 (53.3)	15 (50)	
Permanent molar (*n*%)						0.988
Upper		15 (50)	16 (53.3)	15 (50)	16 (53.3)	
Lower		15 (50)	14 (46.7)	15 (50)	14 (46.7)	

*p value < 0.05 were obtained between VOPI groups based on ANOVA or Chi-square for continuous and categorical variables, respectively, shown as mean ± SD. The non-parametric Kruskal–Wallis H test was used for median and interquartile range (IQR)

A specificity test for all primers was done. For *S. wiggsiae*, the standard strain was not obtained due to the difficulty in laboratory culture. Standard curves were plotted from the quantities and threshold cycle of universal BAC16S, *Bifidobacterium* and *S. mutans* primers. The detection limit of universal BAC16S, *Bifidobacterium* and *S. mutans* primers was 10^3^, 10^1^ and 10^2^, respectively (Fig. 3).

Table 3 shows the prevalence of *S. mutans, Bifidobacterium* and *S. wiggsiae* in each of the VOPI scores. There was no significant difference between prevalence of these bacteria and the VOPI scores. However, the prevalence of *S. mutans* and *S. wiggsiae* increased, whereas *Bifidobacterium* decreased, when the VOPI scores increased.

**Table 3:** Prevalence of *S. mutans*, *Bifidobacterium* and *S. wiggsiae* in four VOPI groups

Bacterial detection	VOPI 0 (*n* = 30)	VOPI 1 (*n* = 30)	VOPI 2 (*n* = 30)	VOPI 3 (*n* = 30)	*p* value
	*n* (%)	*n* (%)	*n* (%)	*n* (%)	
*S. mutans*	29 (96.67)	29 (96.67)	30 (100)	30 (100)	0.064
*Bifidobacterium*	29 (96.67)	29 (96.67)	23 (76.67)	23 (76.67)	
*S. wiggsiae*	9 (30)	19 (63.33)	25(83.33)	30 (100)	

*p value < 0.05, Chi-square tests

Table 4 shows the detection of ICDAS scores in each of the VOPI groups. Amongst samples categorised under VOPI 0, 86.67% showed no caries (ICDAS 0). Thirty three percent of samples in VOPI 3 exhibited enamel caries (ICDAS 3). There is a significant difference between VOPI and ICDAS scores (*p* < 0.001). A severity of enamel caries increased when the VOPI scores increased. Enamel caries severity was observed with higher VOPI scores. Using logistic regression analysis at *p* value < 0.05, results showed that individuals in VOPI 1 were 6.5 times more likely to have higher ICDAS than those in VOPI 0, significantly (*p* = 0.004). Individuals in VOPI 2 were significantly more likely (58.5 times) to have higher ICDAS than those in VOPI 0 (*p* < 0.001).

**Table 4:** ICDAS detection in four VOPI groups

ICDAS detection	VOPI 0 (*n* = 30)	VOPI 1 (*n* = 30)	VOPI 2 (*n* = 30)	VOPI 3 (*n* = 30)	*p* value
	*n* (%)	*n* (%)	*n* (%)	*n* (%)	
ICDAS 0	26 (86.67)	15 (50)	3 (10)	0	< 0.001*
ICDAS 1	4 (13.33)	8 (26.67)	11 (36.67)	6 (20)	
ICDAS 2	0	5 (16.67)	11 (36.67)	14 (46.67)	
ICDAS 3	0	2 (6.67)	5 (16.67)	10 (33.33)	

*p value < 0.05, Chi-square tests

When comparing each of studied bacteria including ratio of *S. mutans* to total bacteria and *Bifidobacterium* to total bacteria between each VOPI scores, as shown in Table 5, results showed that levels of total bacteria count between VOPI scores, the levels of total bacteria count started to show a significant difference at VOPI score 2, but there was no significant difference in the level of total bacteria between VOPI scores 2 and 3. There were significant differences in *S. mutans* quantities between VOPI 0 and VOPI 2 (*p* = 0.018) and between VOPI 0 and VOPI 3 (*p* = 0.049). In addition, a significant difference in *Bifidobacterium* counts between VOPI 0 and VOPI 1 (*p* < 0.001), as well as between VOPI 1 and VOPI 2 (*p* < 0.001) was demonstrated. For the level of ratio of *S. mutans* to total bacteria, it started to show a significant difference at VOPI score 3 (*p* = 0.014), also between VOPI 1 and VOPI 2 (*p* = 0.037), and between VOPI 1 and VOPI 3 (*p* = 0.010). The level of ratio of *Bifidobacterium* to total bacteria started to show a significant difference at VOPI score 2. But there was no significantly different level of total bacteria between VOPI score 2 and 3.

**Table 5:** Comparison of bacteria counts and ratios amongst VOPI groups

	Total bacteria		*S. mutans*		*S. mutans*/total bacteria		*Bifidobacterium*		*Bifidobacterium*/total bacteria	
(I) VOPI	(J) VOPI	*p* value	(J) VOPI	*p* value	(J) VOPI	*p* value	(J) VOPI	*p* value	(J) VOPI	*p* value
0	1	0.912	1	0.511	1	0.819	1	< 0.001*	1	0.072
	2	< 0.001*	2	0.018*	2	0.067	2	0.078	2	< 0.001*
	3	< 0.001*	3	0.049*	3	0.014*	3	0.387	3	< 0.001*
1	2	< 0.001*	2	0.121	2	0.037*	2	< 0.001*	2	< 0.001*
	3	< 0.001*	3	0.277	3	0.010*	3	0.141	3	< 0.001*
2	3	0.169	3	0.657	3	0.574	3	0.120	3	0.188

*p value < 0.05 (Mann–Whitney test), (I) and (J) were for statistics analysis

Levels of total bacteria (*p* < 0.001) and *S. mutans* (*p* = 0.026) were significantly increased when VOPI scores increased. There was a significant difference in the quantities of *Bifidobacterium* amongst VOPI groups (*p* < 0.001). There was a significantly different ratio of *S. mutans* to total bacteria (*p* = 0.015) and *Bifidobacterium* to total bacteria (*p* < 0.001) amongst the VOPI scores (Table 6).

**Table 6:** Analysis of median quantities of all studied bacteria and ratio of *S. mutans*/total bacteria and *Bifidobacterium*/total bacteria

	VOPI 0	VOPI 1	VOPI 2	VOPI 3	*p* value
	Median	Median	Median	Median	
	(Min, Max)	(Min, Max)	(Min, Max)	(Min, Max)	
Total bacteria	1.70 × 10^7^ (3.83 × 10^5^, 1.21 × 10^9^)	1.71 × 10^7^ (4.12 × 10^5^, 1.28 × 10^9^)	1.19 × 10^9^ (4.14 × 10^8^, 2.01 × 10^9^)	1.34 × 10^9^ (6.48 × 10^8^, 2.58 × 10^9^)	< 0.001*
*S. mutans*	5.32 × 10^3^ (0, 4.15 × 10^4^)	6.75 × 10^3^ (0, 3.58 × 10^6^)	2.48 × 10^4^ (3.54 × 10^2^, 4.93 × 10^8^)	3.69 × 10^4^ (2.14 × 10^2^, 1.84 × 10^9^)	0.026*
*S. mutans*/total bacteria	5.03 × 10^–4^ (0, 9.96 × 10^–3^)	2.26 × 10^–4^ (0, 5.98 × 10^–1^)	2.48 × 10^–5^ (1.82 × 10^–7^, 3.29 × 10^–1^)	2.73 × 10^–5^	0.015*
				(8.67 × 10^–8^, 7.13 × 10^–1^)	
*Bifidobacterium*	4.38 × 10^5^ (0, 2.17 × 10^6^)	1.79 × 10^6^ (0, 6.06 × 10^7^)	2.67 × 10^5^ (0, 3.90 × 10^7^)	8.77 × 10^5^ (0, 8.42 × 10^7^)	< 0.001*
*Bifidobacterium*/total bacteria	1.63 × 10^–2^ (0, 5.48 × 10^–1^)	7.49 × 10^–2^ (0, 8.99 × 10^–1^)	2.35 × 10^–4^ (0, 2.98 × 10^–2^)	6.92 × 10^–4^ (0, 3.27 × 10^–2^)	< 0.001*

*p value < 0.05, between VOPI groups based non-parametric Kruskal–Wallis H test

Taken together, levels of *S. mutans* and ratio of *S. mutans* to total bacteria continuously showed the differences until VOPI 3 but not *Bifidobacterium* which showed no difference at VOPI 3.

There were positive correlations between quantities of total bacteria (*p* < 0.001), *S. mutans* (*p* = 0.02), ICDAS scores (*p* < 0.001) and VOPI scores (Table 7). Results showed that amounts of total bacteria and *S. mutans* had increased when VOPI scores increased. In addition, ICDAS scores were higher when VOPI scores increased. On the other hand, there were negative correlations between quantities of ratio of *S. mutans* to total bacteria (*p* = 0.003), also the ratio of *Bifidobacterium* to total bacteria (*p* < 0.001), and VOPI scores and ICDAS scores (*p* < 0.001), indicating that these bacteria ratios were lower when VOPI and ICDAS scores were higher.

**Table 7:** Analysis of the correlation between bacterial levels, VOPI and ICDAS

	Total bacteria		*S. mutans*		*S. mutans/total bacteria*		*Bifidobacterium*		*Bifidobacterium/total bacteria*		ICDAS	
	*ρ*	*p* value	*ρ*	*p* value	*ρ*	*p* value	*ρ*	*p* value	*ρ*	*p* value	*ρ*	*p* value
VOPI	0.743	< 0.001*	0.212	0.02*	–0.271	0.003*	–0.030	0.748	–0.541	< 0.001*	0.724	< 0.001*
ICDAS	0.583	< 0.001*	0.428	< 0.001*	0.029	0.751	–0.125	0.174	–0.492	< 0.001*	1	1

*p value < 0.05, (Spearman’s Rho)

## Discussion

In this study, ICDAS scored 4–6, which indicates dentin caries were not assessed because we aimed to collect samples only at incipient enamel lesions. This is the first study to evaluate the association between VOPI and ICDAS, and our results showed a positive correlation between VOPI and ICDAS (*p* < 0.001).

The structural characteristics and bacterial composition of the biofilm observed within grooves and at the entry points of fissures closely resembled those of supra gingival in vivo biofilms (Zijnge et al. 2010; Schweigel et al. 2016). Plaque formation starts with the salivary pellicle depositing on the tooth surface within 2 h after cleaning. Initial colonisers such as *Streptococcus* species is then detected (Dige et al. 2009; Hannig et al. 2007; Forssten et al. 2010). Increased biofilm acidity correlates with demineralization of the enamel surface, resulting in surface roughening that may facilitate the colonisation of more aggressive microbial pathogens which are secondary colonisers, such as *S. mutans*, *Bifidobacterium* and *Scardovia* species, contributing to climax communities (Forssten et al. 2010; Takahashi et al. 2011). Dige and colleagues examined the biofilm in occlusal grooves and shallow fissures. They discovered that *Streptococcus* species were detected at both intact and non-cavitated carious sites. *S. mutans* was present in both active and inactive caries but absent on sound enamel (Dige et al. 2014).

Most previous studies on the microbiota in occlusal caries have focussed only on dentin caries (Arif et al. 2008; Lima et al. 2011; Mantzourani et al. 2009a, b). Besides *S. mutans*, this study is the first study to measure the amount of novel non-mutans caries-associated bacteria present in occlusal plaque samples collected from caries-free to initial enamel caries on first permanent molars and their association with the VPOI and ICDAS scores. In this study, the prevalence of *S. mutans* was between 96 and 100%. It was detected 100% of the time in the highest VOPI score, which supports the association between *S. mutans* and dental plaque. From previous studies, *S. mutans* was prevalent on occlusal surfaces and has been explored as a risk predictor of the disease (Dinis et al. 2022). In this study, *S. mutans* levels were in the highest VOPI score (VOPI = 3) at 3.69 × 10^4^ CFU/mL, which is in the same range as a previous study using a selective culture-based method and confirmed with a specific monoclonal antibody, showing that *S. mutans* levels in the high caries risk were approximately 10^5^ CFU/mL (Nguyen et al. 2022). Another study was done in adult Musuo people in China, they found the prevalence of *S. mutans* was 75.4% (Wu et al. 2003). Another previous study pooled plaque samples from occlusal surfaces of 87 individual posterior teeth from 30 children in 3 dentition stages (primary, mixed, and permanent). *S. mutans* levels in the occlusal plaque of individual posterior teeth were quantified with quantitative PCR. Results showed that *S. mutans* levels in the occlusal plaque confirmed the preferential colonisation on the first primary and permanent molars (Dinis et al. 2022). In addition, this study demonstrated that amongst samples categorised under VOPI 0, 86.67% showed no caries (ICDAS 0), whereas 33.33% of the samples in VOPI 3 exhibited enamel caries (ICDAS 3). Individuals in VOPI 1 were 6.50 times more likely to have enamel caries compared to those in VOPI 0, whilst individuals in VOPI 2 had a 58.50 times higher chance to have enamel caries than those in VOPI 0. This is consistent with the previous study which found that occlusal surfaces with no or thin occlusal plaque (VOPI 0 or 1) were sound enamel, compared to those with heavy plaque accumulation (VOPI 2 or 3) which were more prone to caries than those with thin (VOPI 1) or no visible plaque (VOPI 0) (Carvalho et al. 2017).

Previous studies reported that *Bifidobacterium* species were exclusively found in active caries sites and in mature plaque from subjects with poor oral hygiene when compared with good oral hygiene subjects (Dige et al. 2014; Mitrakul et al. 2017; Takahashi et al. 2011).

Another previous study obtained plaque samples from sound occlusal surfaces of 12 caries-free adults and 12 children. *Bifidobacterium* were detected using genus-specific PCR primers and were confirmed by 16S rRNA sequencing. Their results showed that no *Bifidobacterium* were isolated from the occlusal surfaces of caries-free adults or children (Mantzourani et al. 2009a, b). Conversely, in this study, the level of *Bifidobacterium* that was detected from VOPI 0 decreased when VOPI scores increased. Our findings showed a different result when compared to the previous study that reported *Bifidobacterium* species were not commonly found within the shallow fissures. We found that the prevalence of *Bifidobacterium* was between 76.67% and 96.67%, which was quite high. We also found that the ratios of *Bifidobacterium* to total bacteria and *S. mutans* to total bacteria were negatively correlated with VOPI scores. Levels of total bacteria increased when the VOPI score increased. Therefore, *S. mutans* and *Bifidobacterium* decreased in ratio. Previous study found that, in cases of active cavitated enamel lesion, *Bifidobacterium* were notably abundant and present in the outer layers of the biofilms at the cavity entrance (Dige et al. 2014). Results from previous studies also found an evident increase in aciduric bacteria associated with dental caries with cavitation, including *S. mutans* and *Bifidobacterium* (Aas et al. 2008; Munson et al. 2004; Celik et al. 2021)]. There is a high possibility that the prevalence of *Bifidobacterium* might increase later on once the enamel breaks down. Several previous studies found *Bifidobacterium* mostly in deep dentinal caries, but rarely found it in intact or white spot lesions (Mantzourani et al. 2009a, b; Becker et al. 2002; Torlakovic et al. 2012; Haukioja et al. 2006). It is possible that *Bifidobacterium* might be associated with caries progression after tooth surface destruction has already begun by other bacteria. The current literature mentions the special properties of *Bifidobacterium* that increase the violence of caries progression because it can store intracellular polysaccharides and degrade them into acids under carbohydrate-limited conditions, such as between meals, and it has tolerance to fluoride due to its unique metabolic pathway (Manome et al. 2019).

*S. wiggsiae* belongs to the *Bifidobacteriaceae* family (Valdez et al. 2016). Previous studies found a significantly higher prevalence of *S. wiggsiae* in the S-ECC group than in the caries-free group (Tantikulchan et al. 2022; Colombo et al. 2016). This study is the first to report the association between *S. wiggsiae* and VOPI scores in children. We found that the prevalence of *S. wiggsiae* (30%) was lower than that of *S. mutans* (96.67%) in the VOPI 0. In the VOPI 3, the prevalence of *S. wiggsiae* increased to 100%, which is equal to the prevalence of *S. mutans*. This suggests that *S. wiggsiae* may be associated with heavy plaque accumulation on the occlusal surface of first permanent molars. Most previous studies reported an association between *S. wiggsiae* and pooled dental plaque from S-ECC children, but not from the occlusal plaque (Matondkar et al. 2020; Tantikulchal et al. 2022; Dhamneonsawat et al. 2024). Previous studies revealed associations between *S. wiggsiae*, *S. mutans* and other acid-producing bacteria with both white spot initial carious lesions and aggressive caries in adolescents (Eriksson et al. 2018; Havsed et al. 2021). *S. wiggsiae* is capable of thriving and producing acid in low-oxygen environments like mature biofilm (Vacharaksa, et al. 2015). It metabolises glucose through a fructose-6-phosphate pathway (F6PPK shunt), producing acetic acid as the end product and lowers the environmental pH to 3.5. Moreover, it demonstrates resistance to fluoride, lactic acid and acetic acid (Kameda et al. 2020). These characteristics contribute to *S. wiggsiae’s* strong ecological competitiveness in acidic environments, thereby exacerbating its cariogenic effects. Both clinical and in vitro studies indicate a symbiotic relationship between *S. wiggsiae* and *S. mutans*. For example, in saliva samples from children, a positive association was observed between *S. wiggsiae* and *S. mutans* (Colombo et al. 2016). Analysis of plaque samples from adolescents revealed an association between *S. wiggsiae* and *S. mutans*, particularly in cases of more aggressive caries. [Eriksson et al. 2018]. Our previous studies also found that subjects who exhibited all three bacteria (*S. mutans* + *Bifidobacterium* + *S. wiggsiae*) had higher dmft scores in S-ECC children (Tantikulchal et al. 2022; Dhamneonsawat et al. 2024). This study focusses on biofilms in relation to occlusal caries especially during non-cavitated lesions or incipient enamel lesions help better understanding the role of occlusal biofilms and related bacterial species in caries development which eventually helps to evaluate the risk for occlusal caries lesion activity, incidence, and progression clinically in the future, in the hope to develop a tool to assess the lesion activity status to determine the non-invasive treatment need.

Limitations of this study are that it is a cross-sectional design which provides only a snapshot in time and does not allow for determination of the true dynamics of the oral biofilm community. In addition, samples are limited to only one area in the central part of Thailand, which might not represent the entire population.

## Conclusion

Within the limitations of the present cross-sectional study, it has been shown that ages of the children were significantly different amongst VOPI groups. The prevalence of *S. mutans* and *S. wiggsiae* increased, whereas *Bifidobacterium* decreased, when the VOPI scores increased. Quantities of total bacteria, *S. mutans*, ICDAS scores and VOPI scores were positively correlated. Quantities of ratio of *S. mutans/*total bacteria, ratio of *Bifidobacterium*/total bacteria (*p* < 0.001), VOPI scores and ICDAS scores were negatively correlated. Our study demonstrated that these bacteria were associated with occlusal biofilm and incipient enamel caries in children.
